# Visitor responses to emergency evacuation: A human behavior approach

**DOI:** 10.34172/hpp.025.44366

**Published:** 2025-12-30

**Authors:** Omid Akbarzadeh, Parisa Moshashaei, Hamed Golzad, Henry Liu, Seyed Shamseddin Alizadeh

**Affiliations:** ^1^Department of Occupational Health and Safety, Faculty of Health, Tabriz University of Medical Sciences, Tabriz, Iran; ^2^School of Built Environment and Design, Faculty of Art and Design, University of Canberra, Canberra 2617, Australia

**Keywords:** Social behavior, Emergency shelter, Disaster planning, Safety management, Decision making, Urban health, Crowding

## Abstract

**Background:**

This study investigates human behaviours during emergency evacuations using data collected at the Tabriz Grand Bazaar (TGB), a UNESCO World Heritage site and the world’s largest covered market. While past studies modelled crowd evacuations through simulations, limited empirical research exists on real human behaviour.

**Methods:**

Drawing on 749 questionnaire responses from TGB visitors, the study explores four key aspects of evacuation dynamics: (1) proactive vs. reactive responses; (2) competitive vs. cooperative interactions; (3) symmetry-breaking behaviours; and (4) route selection.

**Results:**

The analysis revealed that 44.2% of participants reported engaging in competitive behaviours (e.g., pushing), while only 8.4% showed a strong willingness to help others. Over 90% tended to follow others during evacuations, and 77.8% preferred to avoid the least crowded exit, indicating high symmetry-breaking tendencies. No significant correlation was found between gender and evacuation behaviours, but marital status, education, and age were significantly associated with different behavioural strategies.

**Conclusion:**

The study contributes by (1) providing behavioural insights for emergency planning, (2) informing the design of realistic evacuation simulations, and (3) offering empirical evidence to support future research.

## Graphical Abstract

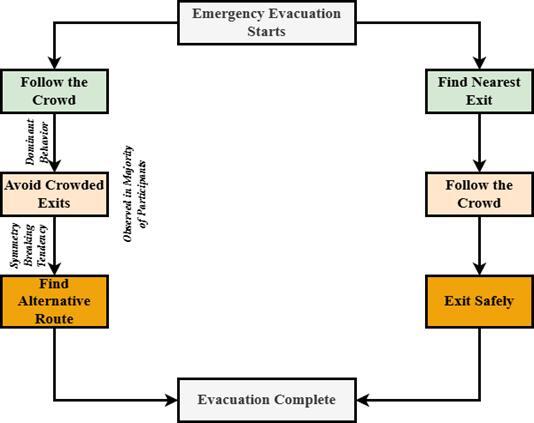

## Introduction

###  Background

 Ensuring the safety and managing the behavior of crowds during emergencies in public areas has been acknowledged as being a significant challenge. A case in point is the bazaar, which has been a central hub for commerce in Middle Eastern cities, serving as a crucial component of urban life.^1^ For example, the Tabriz Grand Bazaar (TGB), listed as a UNESCO World Heritage site and recognized as the world’s largest covered market, serves as an economic, social, religious, communicative, political, and cultural hub for the local people.^2^ Therefore, crowd dynamics and pedestrian flow are key areas of study within the context of emergency evacuations. Past incidents, such as the Plasco building fire in Tehran in 2017^3^ and the Baku subway fire in Azerbaijan in 1995 resulted in 22 and 289 fatalities and 70 and 270 injuries, respectively. A main reason for ineffective evacuations during such disasters is a limited understanding of how people are likely to behave in emergency situations. Research in this field emphasizes the importance of training and preparedness, which can significantly improve response times and outcomes in real-world emergencies.^4^ Additionally, effective evacuation procedures are often informed by the principles of crowd psychology and behavior, which help predict how individuals and groups are likely to act under stress.^5^

 Computational models and simulations have been widely used to analyze and optimize evacuation strategies. These models consider variables such as exit availability, crowd density and individual decision-making processes, providing insights that can enhance safety protocols.^6^ Fundamentally, they can be classified into micro and macro approaches.^7^ Additionally, simulations have been employed to develop and test emergency evacuation plans.^8^ The micro-level models focus on the behaviors and interactions of individual pedestrians.^9^ They focus on capturing the dynamics of an individual pedestrian’s movement, considering factors such as personal space, speed, direction, and decision-making in response to the surrounding environment, whereas macro-level models examine the collective movement patterns of large pedestrian groups,^10^ abstracting away individual behaviors to address the overall flow and distribution of the crowd. They are concerned with the emergent properties of pedestrian movement and crowd dynamics in large-scale scenarios like urban traffic or evacuation processes.^11,12^ The macro-level models provide a broader perspective, emphasizing the aggregate effects of many individuals moving together rather than the specifics of individual interactions.^13^ However, Shiwakoti et al argue that the current models, including the micro-level and macro-level approaches, fall short of accurately representing panic behavior and the influence of environmental and social factors.^14^

 Advanced computational techniques, such as agent-based modeling^15^ and discrete event simulation,^16^ are normally adopted to enhance the accuracy of the micro and macro level models.^17^ However, there remains a critical need for a comprehensive empirical data, particularly in emergency situations, to uplift the reliability of evacuation models. Current models do not fully capture the complexity of human behavior in such situations as panic, decision-making under pressure or the influence of others, leading to less reliable predictions in emergency situations.^18-20^ Collecting detailed empirical data from real-life scenarios, experiments, and monitoring technologies is crucial for refining and validating simulation models, enabling more accurate representation of real-world conditions and enhancing evacuation strategies.^21^

 Several studies have conducted surveys of passengers in public spaces to examine operational features, such as safety concerns related to crowding behavior, waiting times, and pre-evacuation durations.^22^ These surveys provide key insights into crowd perception and behaviour, supporting hazard identification and, when combined with simulations and real-world data, help develop more reliable evacuation models.^23^

 Despite the increasing use of computational models and simulations to study emergency evacuations, there is a lack of empirical studies that examine real-world human behavior in such situations, particularly in complex, high-density environments like traditional marketplaces. Many Empirical research on real-world evacuation behavior in complex, high-density settings like traditional marketplaces is scarce, with existing models often overlooking socio-cultural factors and decision-making under pressure. The TGB, with its irregular layout, dense foot traffic, and diverse visitors, offers a unique case for addressing this gap and improving evacuation planning.

 While numerous studies have explored emergency evacuations through mathematical simulations and theoretical models, there is a significant gap in empirical research that captures real human behaviours during emergencies, especially in complex, high-density environments like traditional marketplaces. Previous models often assume idealized conditions and overlook sociocultural factors, individual decision-making under stress, and spatial complexity. In contrast, this study directly addresses that gap by empirically examining evacuation behaviours at the TGB, using real-world data from 749 participants. By doing so, it contributes practical insights into four critical behavioural dimensions, proactive vs. reactive responses, competitive vs. cooperative interactions, symmetry-breaking behaviours, and route selection, thereby enhancing both theoretical understanding and the realism of future evacuation models. 

###  Study Goals

**Figure 1:**
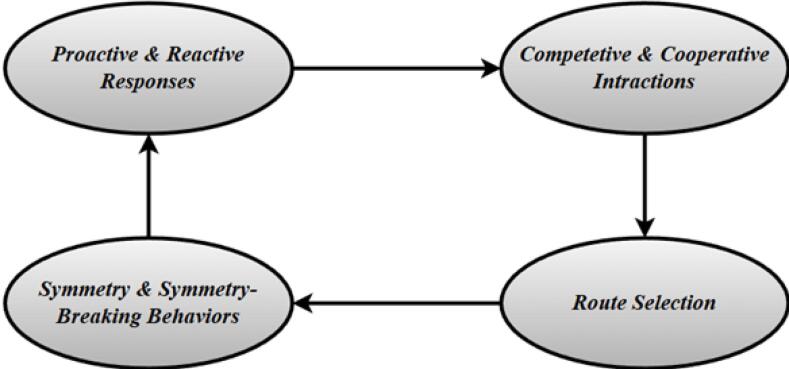
Conceptual framework of key strategies in emergency evacuation dynamics

 Although simulations have advanced the study of crowd evacuation, they often overlook the socio-cultural complexity of real settings such as the TGB, where established theoretical patterns may not fully apply. To address this gap, the present study investigates the behavioural dynamics that emerge during emergency evacuation in a live, historically significant environment. The first goal is to determine whether visitors display a stronger inclination toward proactive behaviours, such as taking early action or seeking alternative routes, rather than reactive responses triggered by external cues or delayed decision-making. The second goal is to analyse whether competitive behaviours, driven by urgency and self-preservation, are more dominant than cooperative actions that involve mutual support and collective movement. A further aim is to characterise the strong tendency toward symmetry-breaking behaviour, characterised by a disproportionate preference for particular exits despite equivalent accessibility. This behavioural shift often arises due to social following, perceived safety cues, or panic, and can lead to crowding, queue imbalance, and evacuation delays. Finally, the study examines whether demographic factors, including age, education, and marital status, significantly shape evacuation behaviours, while gender is expected to show a limited effect in this context. Figure 1 illustrate the key strategies in emergency evacuation plan. By focusing on the TGB as a case study, the research offers context-specific insights that enhance both theoretical models and practical strategies for emergency management in complex environments.

## Theoretical Framework

 Reactions of different people to emergency situations can vary from being more reactive to more proactive.^4^ In certain situations, individuals responded reactively when instructions were given over the public address system or received from staff members. Conversely, in other scenarios, people adopted a proactive approach, quickly moving to exits and utilizing emergency call switches without waiting for instructions.^24^ Some studies have demonstrated that during emergencies, individuals moving towards exits exhibit behaviors such as pushing and assisting others.^25^ A competitive approach has been linked to slower evacuation rates and longer waiting times, potentially leading to severe outcomes such as crushing and stampedes. Additionally, research on mass disasters has revealed that some individuals tend to follow others and ignore available alternative exits during emergencies, reflecting a tendency towards symmetry-breaking behavior.^26^ Mathematical models predict that during emergencies, individuals may follow others instead of using optimal routes, causing uneven exit use. These models, which consider crowd density, perceived safety, and social influence, help explain why people often cluster at specific exits.^27^

###  Proactive and Reactive Responses

 Emergency evacuations rely on both proactive and reactive strategies. Proactive measures, like risk assessments, hazard identification, and evacuation drills, enhance preparedness and have been shown to reduce evacuation time and increase compliance during real incidents.^28^ Also, proactive measures such as installing emergency buttons, establishing evacuation paths, and providing emergency exits promote a sense of readiness and situational awareness among people and organizations.^29^

 In the context of the TGB, proactive behaviour might be exemplified by a visitor who quickly assesses their surroundings during an emergency, identifies the nearest visible exit, and proceeds without waiting for instructions. For instance, a shopkeeper familiar with the bazaar’s layout may immediately activate an emergency button and direct nearby visitors toward safe exits. Conversely, reactive behaviour could involve a tourist or first-time visitor who remains stationary during an emergency, waiting for guidance from public address announcements or assistance from security personnel. These examples illustrate how individuals’ familiarity with the environment and perceived authority influence their evacuation strategies.

 Contrarily, reactive reactions involve decision-making, and actions taken during the circumstance, concentrating on real-time adaptations to extending conditions. Investigation underlines that sufficient reactive reactions depend on communication, supervision, and the ability to manage crowd dynamics under stress.^30,31^ While reactive plans are crucial in unexpected scenarios, proactive planning can decrease the dependence on reactive measures, enhancing overall evacuation outcomes. Balancing both strategies is vital for comprehensive emergency evacuation management, as proactive strategies improve preparedness, while reactive reactions ensure flexibility in emergencies.

###  Competitive and Cooperative Interactions

 Emergency evacuations often involve a mix of cooperative and competitive behaviors that influence both safety and speed. In high-stress situations, individuals may act competitively by pushing or rushing at bottlenecks, which can lead to crowd turbulence and increase the risk of injury. Helbing et al describe this as panic-induced behavior, where competitive dynamics escalate the likelihood of harm.^32^ Such behavior is often intensified by anxiety, limited time, and insufficient crowd management, leading to chaotic and unsystematic evacuation procedures.^33^

 On the other hand, cooperative behavior plays a critical role in promoting softer and safer evacuations. Cooperation can manifest as people helping others, following social norms, and adhering to guidelines from emergency personnel. Drury et al claim that cooperative behavior is often caused by social originality, where people perceive themselves as part of a crowd encountering a shared threat.^34^ This “collective resilience” can promote joint support and decrease panic. Bode et al also highlight that trust in leaders and clear communication are essential in fostering cooperative behavior, as evacuees are more likely to assist each other and follow procedures when they feel educated and reassured.^35^

###  Symmetry and Symmetry-Breaking Behavior

 Symmetry in emergency evacuation refers to the even and balanced use of available exits, where individuals are equally distributed across multiple egress options, resulting in optimal flow and minimal congestion. Symmetry-breaking behavior, in contrast, occurs when this balance is disrupted, individuals disproportionately prefer one or a few exits, even when others are available.^36^ This deviation is often influenced by environmental factors (e.g., visibility, signage), psychological cues (e.g., panic or stress), or social pressures. While symmetry-breaking may resemble herding behavior, defined as the tendency to follow others without independent judgment, the two differ in nuance.^37^ Herding behavior emphasizes conformity based on social cues or the presence of others, while symmetry-breaking focuses on the structural imbalance that emerges in exit usage, often because of herding.^38^ The implications for evacuation efficiency are critical: symmetry-breaking can lead to severe bottlenecks, underutilization of safer or faster routes, and delayed evacuation times.^39^ As such, understanding and mitigating these tendencies through design and communication strategies is essential for improving emergency egress outcomes.

 Investigations have shown that symmetrical evacuation dynamics are more reasonable when emergency door capacities are well-matched to mass density, and guidance procedures provide consistent information across the environment.^40^ Computational models often integrate hypotheses of symmetry to simulate ideal evacuation strategies, emphasizing the significance of spatial design in fostering balanced crowd movement.

 However, symmetry-breaking behavior is expected in real-life emergencies because of psychological, social, and environmental factors. Asymmetrical flow emerges when individuals disproportionately prefer specific exits, often due to stress, herding manners, or miscommunication.^41^ For example, Moussaïd et al demonstrated that in dense crowds, people often follow others in a herd-like behavior, resulting in congestion at certain exits.^42^ Environmental characteristics, such as inadequate signage, exit obstructions, and irregular exit visibility, additionally contribute to symmetry-breaking.^43^

###  Route Selection

 Route choice in emergencies is shaped by environmental factors like visibility, distance, and obstacles, as well as psychological and social influences such as panic, familiarity, and crowd behavior, all of which affect speed, safety, and effectiveness.^44^ Recent investigations indicate that in high-stress scenarios, people frequently prefer familiar or visually major routes, even if alternative pathways are more efficient.^45^ This preference underscores the importance of designing environments with intuitive layouts that guide people to optimal emergency exits. As Cuesta et al note, effective wayfinding can significantly improve route selection by reducing chaos and uncertainty.^46^

 Social factors play a crucial role in shaping route selection, specifically in crowded environments. Herding behavior, where people follow others rather than make self-reliant decisions, is a comprehensively detailed phenomenon in emergency evacuations.^47^ Wang et al emphasize that real-time communication systems, including digital displays and audio announcements, can help manage herding by directing people toward less crowded routes.^48^ Evacuation training and exercises further improve decision-making by introducing people to alternative routes and facilitating adaptive behavior in dynamic conditions.^49^

## Methods

###  Data Collection

 A questionnaire survey, inspired by Nirajan Shiwakoti et al’s study, was designed to examine the potential behaviors of passengers during an emergency in TGB. The bazaar sees an average of over 20,000 visitors on weekdays.^50^ The TGB’s complex layout, dense crowds, and diverse visitors make it a valuable setting for studying real-world evacuation behavior in irregular, high-density environments. Located in Tabriz’s Central Business District, the bazaar attracts a wide demographic, and this study was conducted with full ethical approval and informed consent from participants.

 Prior to data collection, the study received formal ethical approval from the Tabriz University of Medical Sciences Ethics Committee. Additionally, permissions were obtained from the Tabriz Municipality and the administrative board of TGB to ensure smooth field operations. The study included visitors aged 15 years and older who were able to understand and respond to the questionnaire, while excluding those who were illiterate, under 15 years old, or had cognitive impairments that could affect their ability to answer behavioral questions accurately.

 The survey was conducted between 12:00 PM and 8:00 PM to capture peak-hour visitor diversity, with voluntary participation following informed consent and provision of study details. Data integrity was supported through trained surveyors using neutral questioning, assurances of anonymity and confidentiality, and a pilot test with 100 respondents that led to minor wording refinements for clarity.

 A pilot test involving 100 visitors, selected through stratified random sampling to reflect diversity in age, gender, and visit purpose, was conducted to ensure the questionnaire’s clarity and reliability. The sample had a balanced gender distribution (52% male, 48% female), ages ranging from 15 to 65 years (mean 37.4), and varied visit purposes (shopping 50%, work 28%, tourism/leisure 22%). Participants completed the survey and provided feedback on clarity, relevance, and wording, leading to rephrasing of ambiguous items, simplifying technical terms, and replacing confusing terms (e.g., “master point” with “assembly point” in Persian). Long questions were split into shorter items, and the order of questions was refined for better logical flow and reduced fatigue. These improvements increased comprehension, completion rates, and internal consistency (Cronbach’s alpha = 0.87), enhancing the reliability and validity of the main study data.

###  Sampling Strategy

 To ensure a representative sample of TGB visitors, a stratified random sampling approach was employed. This method divides the population into subgroups based on demographic characteristics and visit purposes, ensuring that key groups are adequately represented. The sampling framework consisted of the following strata:

1. Age groups: (a) 15–30 years, (b) 31–50 years, (c) 51 + years
2. Gender: Male and female
3. Education level: (a) High school or below, (b) University degree
4. Visit purpose: (a) Shopping, (b) Work, (c) Tourism/Leisure
5. Familiarity with the Bazaar layout: (a) Frequent visitors (weekly), (b) Occasional visitors (monthly), (c) First-time visitors

The stratification ensured that the final sample reflected the diversity of TGB visitors, addressing potential biases that might arise from an overrepresentation of certain groups. The study aimed for a sample size of approximately 750 participants, following standard guidelines for survey-based behavioral studies in high-density environments. This sample size was determined based on power analysis, ensuring sufficient statistical power to detect significant behavioral trends across different demographic groups.

###  Survey Design

 The survey included 14 validated behavioral questions, organized into four categories of passenger behavior during emergencies and rated on a 5-point Likert scale (1 = “Very Unlikely” to 5 = “Very Likely”). This scale was chosen for its proven reliability, ease of use, and suitability for high-traffic settings like the TGB, with pilot testing confirming clarity and high internal consistency. Demographic data (gender, age, education) were also collected to analyze variations in emergency responses across different population groups, enhancing insights for improving evacuation strategies and safety protocols. Passenger behaviors were classified into six categories, proactive (e.g., moving to doorways, using emergency keys, calling emergency services, going to assembly points), reactive (waiting for announcements, personnel, or at assembly points), competitive (shoving others), cooperative (helping others), symmetry-breaking (following others, choosing less crowded exits), and route choice (using elevators, escalators, tunnels, or staircases). These categories, based on prior studies, were assessed using a 5-point Likert scale and validated through a pilot test with 100 participants. Ordinal logistic regression was then applied to examine how demographic factors such as age, gender, marital status, education, visit purpose, and familiarity with the bazaar influenced evacuation decision-making patterns.

###  Data Analysis Methods

 To assess the likelihood of a particular behavior, we conducted both a one-sample t-test and its non-parametric equivalent for each scenario using STATA 17 software package. In addition, we calculated the frequency, mean and standard deviation of the observed data as well as evaluating the statistical significance of the relationships between variables under *P *values. This approach enables quantifying and analyzing the behavior in a robust manner through considering both parametric and non-parametric perspectives to ensure the validity and reliability of our findings.

 To ensure data quality, questionnaires with more than two missing key items were excluded, uniform response patterns were flagged, and question order was randomized to reduce bias. Where possible, surveys were self-administered to limit interviewer influence, and participants were informed there were no “right” or “wrong” answers to minimize social desirability bias.

 Additionally, to assess the effectiveness and significance of different evacuation and emergency exit plans, paired sample t-tests and their non-parametric equivalents were carried out. The statistical analysis was guided by two hypotheses: the null hypothesis assumed no difference between the effectiveness of the two strategies, while the alternative hypothesis proposed that one strategy is superior. This approach offered a rigorous framework for determining which evacuation plan may provide greater safety and efficiency in emergencies.

 All *P *values are reported consistently using standard thresholds, such as “*P* < 0.001,” to reflect conventional reporting practices in behavioural sciences. Although multiple statistical tests were performed, no formal correction for multiple comparisons (e.g., Bonferroni adjustment) was applied. Therefore, results should be interpreted with caution, particularly where p-values approach the significance threshold.

 To examine how demographic factors influence behavioral responses, several ordinal logistic regressions were performed. This method was appropriate given the use of a 5-point Likert scale, allowing for consistent and efficient estimates. The findings support the development of evacuation plans and safety protocols that address the diverse needs of the population, thereby improving emergency response effectiveness. The statistical methods used, one-sample t-tests, paired-sample t-tests, and ordinal logistic regression, were selected based on the data structure and research goals. One-sample t-tests assessed deviations from neutral behavior, while paired-sample t-tests compared different strategies. Ordinal logistic regression was applied to capture the effects of demographic variables on Likert-scale responses, ensuring precise and reliable analysis of behavioral patterns.

**Figure 2:**
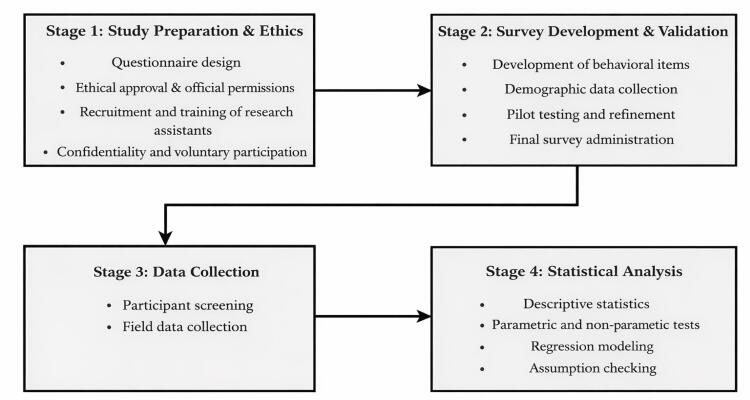
Flow diagram of research method

 When conducting an ordinal regression analysis, it is essential to test the assumption of parallel lines. The proportional odds assumption, also known as the parallel line’s assumption, is essential in ordinal regression. It states that the effect of predictor variables remains constant across all thresholds of the ordinal outcome. This means the relationship between predictors and the odds of being in a higher versus lower category is consistent, allowing the model to use a single set of coefficients for all outcome levels. Finally, Comparative analyses were performed to assess the influence of demographic factors on participants’ responses. To achieve this, a chi-square test was applied to categorical variables,^51^ while a t-test was utilized for continuous variables that followed a normal distribution. Figure 2 presents the steps undertaken to fulfil the aim of this study.

## Results

###  Participants 

 Out of 760 collected responses, 11 were excluded due to missing demographic or behavioral data, resulting in 749 complete questionnaires used for analysis. This filtering ensured data quality and strengthened the reliability of conclusions about evacuation behavior. The data cleaning process involved several steps to ensure the quality of the dataset:

- All collected responses were initially screened for completeness as addressed above. Any questionnaire with missing answers for more than two behavioral questions or lacking demographic information such as age or gender was marked for exclusion.
- For questionnaires with only one or two missing responses, imputation techniques such as mean substitution or hot-deck imputation has been employed in previous studies.^52^ However, to maintain the highest data quality, these questionnaires were ultimately excluded.
- The remaining responses were further verified to ensure that they met the inclusion criteria. Responses that showed inconsistencies or patterns suggesting non-serious answers were also excluded.

The final dataset of 749 complete responses provided a robust foundation for the analysis. It was entered to a comprehensive statistical analysis to unpack the factors influencing evacuation dynamics and examine the behavioral models.

 Basic descriptive statistics were calculated to summarize the demographic characteristics of the participants, such as age distribution, gender distribution and education levels (Table 1).

**Table 1:** Demographic characteristics of participants (n = 749)

**Variable**	**Category**	**Frequency (n)**	**Percent**
Gender	Male	412	55.0
	Female	337	45.0
Age group	15–30 years	311	41.5
	31–50 years	286	38.2
	51 + years	152	20.3
Education level	High school or below	345	46.1
	University degree	404	53.9
Marital status	Single	369	49.3
	Married	380	50.7
Purpose of visit	Shopping	382	51.0
	Work	197	26.3
	Tourism/Leisure	170	22.7
Familiarity with the Bazaar	Frequent visitors (weekly)	246	32.8
	Occasional visitors (monthly)	298	39.8
	First-time visitors	205	27.4

###  Findings

Table 2 summarizes the likelihood of various evacuation strategies reported by participants. The most frequently endorsed behaviors included moving to the assemblypoint, calling emergency services, using the emergency button, waiting at the assemblypoint, following others, and heading to exits. In contrast, using elevators, escalators, and selecting the least crowded exit were among the least likely actions, suggesting a general tendency toward conventional or guided evacuation methods.

**Table 2:** Summary of possible strategies

**Variables**		**Very unlikely** **frequency** **(valid percent)**	**Unlikely** **frequency** **(valid percent)**	**Neutral** **frequency** **(valid percent)**	**Likely** **frequency** **(valid percent)**	**Very likely** **frequency** **(valid percent)**
Proactive strategy	Move to exit	27 (3.6)	54 (7.2)	59 (7.9)	302 (40.3)	307 (41)
	Use emergency bottom	52 (7)	96 (12.7)	111 (14.8)	317 (42.4)	173 (23.1)
	Call emergency	26 (3.5)	57 (7.6)	64 (8.4)	272 (36.4)	329 (44)
	Go to assembly point	20 (2.7)	29 (3.9)	49 (6.9)	266 (35.5)	385 (51.4)
Reactive strategy	Wait for PA	83 (11.1)	125 (16.7)	137 (18.3)	255 (34)	149 (19.9)
	Wait for station staff	111 (14.8)	166 (22.2)	179 (23.9)	224 (29.9)	69 (9.2)
	Wait at assembly point	27 (3.6)	61 (8.1)	87 (11.6)	317 (42.3)	257 (34.3)
Competitive strategy	Push other passengers	219 (29.2)	147 (19.6)	100 (13.4)	168 (22.4)	115 (15.4)
Cooperative strategy	Help other people	121 (16.2)	185 (24.7)	194 (25.9)	186 (24.8)	63 (8.4)
Symmetry breaking strategy	Follow other passengers	23 (3.1)	34 (4.6)	83 (11.1)	312 (41.8)	295 (39.5)
	Choose least crowded exit	418 (55.8)	165 (22)	61 (8.1)	72 (9.6)	33 (4.4)
Route Choice Strategies	Use lift	313 (41.8)	182 (24.3)	98 (13.1)	104 (13.9)	52 (6.9)
	Use escalator	274 (36.6)	165 (22.1)	145 (19.4)	110 (14.7)	54 (7.2)

 Behavioral consistency was observed in responses to items like “helping others” and “waiting for station staff,” while behaviors such as “going to the assembly point” and “choosing the least crowded exit” showed more polarized responses. This pattern reflects varying degrees of decision certainty across different behavioral types.

 Paired-sample t-tests indicated that participants consistently preferred one behavior over its alternative, rejecting the null hypothesis at the 99% confidence level. This suggests a clear inclination toward proactive behaviors aimed at enhancing safety.

 Gender was found to have no statistically significant effect on behavioral choices, whereas age and other demographic factors showed meaningful associations, as confirmed by ordinal logistic regression models (Tables 3 and 4).

**Table 3:** Relationship between evacuation strategies and gender and martial situation

	**Gender**				**Martial situation**					
	**Man**	**Woman**	**Total**	***P*** ** value**	**Effect size**	**Single**	**Married**	**Total**	***P*** ** value**	**Effect size**
	**Mean±SE**	**Mean±SE**	**Mean±SE**			**Mean±SE**	**Mean±SE**	**Mean±SE**		
Age	35.65 ± 0.56	29.03 ± 0.57	33.58 ± 0.44	< 0.001	11.72	25.84 ± 0.37	40.18 ± 0.57	33.58 ± 0.44	< 0.001	29.84
Proactive strategy	75.96 ± 0.82	74.57 ± 1.17	75.53 ± 0.67	0.336	1.38	73.36 ± 0.97	77.36 ± 0.92	75.53 ± 0.67	0.003	4.23
Reactive strategy	59.89 ± 1.03	62.11 ± 1.36	60.59 ± 0.82	0.211	- 1.84	58.84 ± 1.14	62.08 ± 1.17	60.59 ± 0.82	0.050	2.8
Competitive strategy	41.72 ± 1.6	48.2 ± 2.41	43.76 ± 1.34	0.024	- 3.17	50.58 ± 1.97	37.96 ± 1.78	43.76 ± 1.34	< 0.001	- 6.72
Cooperative strategy	62.38 ± 0.91	60.85 ± 1.3	61.9 ± 0.75	0.343	1.36	58.72 ± 1.11	64.61 ± 0.99	61.9 ± 0.75	< 0.001	5.6
Symmetry breaking strategy	29.12 ± 1.21	26.2 ± 1.57	28.2 ± 0.96	0.160	2.08	25.15 ± 1.24	30.78 ± 1.42	28.2 ± 0.96	0.004	4.22
Route choice strategy	32.18 ± 1.1	29.35 ± 1.38	31.41 ± 1.02	0.240	2.27	27.42 ± 1.18	33.61 ± 1.54	31.41 ± 1.02	0.002	4.51

**Table 4:** Relationship between evacuation strategies and education level and age group

	**Education Level**				**Age Group**					
	**High school**	**Universities**	**Total**	***P*** ** value**	**Effect size**	**15-30**	**30-60**	**Total**	***P*** ** value**	**Effect size**
	**Mean±SE**	**Mean±SE**	**Mean±SE**			**Mean±SE**	**Mean±SE**	**Mean±SE**		
Age	36.94 ± 0.66	29.62 ± 0.48	33.58 ± 0.44	< 0.001	-12.68	NA	NA	NA	NA	NA
Proactive strategy	74.86 ± 0.96	76.31 ± 0.93	75.53 ± 0.67	0.282	1.53	72.57 ± 0.98	78.02 ± 0.91	75.52 ± 0.67	< 0.001	5.76
Reactive strategy	60.93 ± 1.11	60.19 ± 1.23	60.59 ± 0.82	0.655	- 0.63	57.77 ± 1.17	62.92 ± 1.15	60.57 ± 0.83	0.002	4.44
Competitive strategy	40.53 ± 1.79	47.54 ± 1.99	43.76 ± 1.34	0.009	3.7	50.22 ± 1.98	38.4 ± 1.79	43.81 ± 1.35	< 0.001	- 6.26
Cooperative strategy	64.57 ± 1.01	58.75 ± 1.09	61.9 ± 0.75	< 0.001	- 5.54	59.6 ± 1.11	63.93 ± 1.01	61.94 ± 0.75	0.004	4.08
Symmetry breaking strategy	31.19 ± 1.4	24.69 ± 1.27	28.2 ± 0.96	0.001	- 4.86	23.72 ± 1.21	32.13 ± 1.44	28.29 ± 0.97	< 0.001	6.32
Route choice strategy	30.64 ± 1.15	23.88 ± 1.34	31.41 ± 1.02	< 0.001	- 5.41	24.56 ± 1.13	31.48 ± 1.71	31.41 ± 1.02	< 0.001	4.77

 As shown in Table 3, gender does not significantly influence most evacuation behaviors, except for a competitive strategy involving pushing, where a notable gender difference was observed. Marital status, however, was significantly associated with all strategies examined, with a particularly strong link to symmetry-breaking behavior, indicating its important role in shaping responses. According to Table 4, education level had no significant effect on proactive or reactive behaviors. In contrast, age demonstrated a strong and consistent association with all behavioral strategies, suggesting it is a key determinant of decision-making during emergencies.

## Discussion

 This study examined key behavioral strategies during emergency evacuations, including proactive versus reactive responses, competitive versus cooperative interactions, and symmetry-breaking behavior. The results revealed a dominant preference for proactive strategies, limited cooperative responses, and noticeable symmetry-breaking tendencies. These findings support the study’s aim of deepening our understanding of crowd behavior in complex environments.

 While some gender-based variations were observed in reactive responses, statistical analyses did not find a significant overall relationship between gender and the types of strategies used. This suggests that although men and women may behave differently in emergencies, gender alone is not a strong predictor of strategy selection. Other factors, such as age or situational context, may play a more influential role. This contrasts with prior studies, such as Ekenga and Ziyu,^53^ which found that women tend to exhibit more reactive behaviors in emergency scenarios.

 The absence of significant gender differences in evacuation behavior in our study contrasts with findings from other contexts, where women have been shown to engage more frequently in cooperative or risk-averse strategies. This discrepancy may reflect the influence of sociocultural factors unique to the Iranian context, where public behaviors, particularly in mixed-gender settings, are shaped by cultural expectations and norms.^54^ It is possible that social pressures or gender role internalization led to more convergent behaviors between men and women in public environments such as the TGB.^55^ These findings underscore the importance of interpreting evacuation behavior within a cultural framework and highlight the need for more cross-cultural studies to understand how local norms and values shape decision-making during emergencies. The study observed that women’s approach tends to be more immediate and involved compared to other groups, indicating a heightened level of responsiveness during critical moments.^53^ The situation would have been different if women had taken a more proactive stance. The variation between the studies could be due to such sociocultural and socioeconomic factors as child care responsibilities, poverty, social networks, and ethnic beliefs and discrimination.^56^

 Further analysis of gender differences shows that while both men and women favor proactive strategies, women are more inclined toward reactive behaviors, often seeking help after an emergency escalates rather than initiating early contact with emergency services. This trend highlights the need for further investigation into underlying factors such as risk perception, decision-making patterns, and social influences that may shape these gendered responses.

 The significant relationship between age, education, and behavioral responses underscores the importance of targeted interventions. Younger participants and those with higher education levels showed greater competitive tendencies, possibly reflecting more individualistic approaches under pressure. This finding is consistent with previous research suggesting that these groups may prioritize efficiency over collaboration during emergencies.^57^ Recognizing these patterns can guide the design of demographic-specific training programs that promote cooperative strategies.

 The Zurich Metro fire incident offers valuable insight into the influence of passenger behavior on emergency outcomes. Despite existing protocols and signage, a delayed evacuation occurred until an emergency button was activated, which then triggered a coordinated response. This case highlights the critical role of human decision-making, the design of emergency systems, and the importance of training and preparedness. These findings parallel the themes of the current study, emphasizing the need to consider behavioral factors when developing evacuation strategies.^58^

 The time required to evacuate a confined space during an emergency is crucial for protecting lives. Numerous mathematical models have proposed various theories on this issue.^59,60^ The findings suggest that effective emergency response heavily relies on proper training of station staff, particularly in implementing reactive strategies. Clearly defined roles and responsibilities are essential for efficient emergency management. Furthermore, urban infrastructure planners should prioritize the availability of functional emergency call buttons and ensure that options such as contacting emergency services or moving to assembly points are clearly accessible in crowded public areas.

 Based on past research on crowd disasters, people’s behavior during emergencies can vary. When faced with a crisis, people might adopt different strategies to handle the situation. In some cases, individuals may work together harmoniously to manage the emergency. This could involve helping each other, sharing resources, and coordinating efforts to ensure everyone’s safety.^61^ A study by Cheng and et al explored how cooperative and competitive behaviors influence evacuation during emergencies. They found that people who show cooperative behaviors tend to evacuate more efficiently. These findings resonate with the results of this research, highlighting the importance of cooperation in such critical situations.^62^

 The relatively low prevalence of cooperative behaviour (8.4%) can be better understood through the lens of Social Identity Theory,^63^ which posits that individuals are more likely to engage in prosocial or altruistic actions when they perceive themselves as part of a shared social group. In the context of the TGB, many visitors may have viewed others as strangers, lacking a salient group identity that would encourage mutual aid. This lack of perceived group belonging may have contributed to the low levels of cooperative behaviour observed.^64^ Prior research similarly suggests that a shared sense of identity during emergencies enhances solidarity and supportive actions.^65,66^

 In some situations, individuals may adopt self-interested or competitive behaviors, prioritizing their own safety even at the expense of others. Such actions can result in conflict or physical pushing during evacuations. The choice between cooperative and competitive strategies is often shaped by factors such as the type of emergency, surrounding social cues, and individual personality traits.^67^ The statistical analysis found no general tendency toward competitive behavior among participants. Although gender was not significantly associated with competitiveness, women showed higher levels of cooperation than men. Additionally, participants under 30 and those with a university education exhibited more competitive tendencies during emergencies. These findings suggest that age and educational background may influence whether individuals adopt cooperative or competitive behaviors in high-pressure situations.^68^

 Approximately 44.2% of participants reported engaging in pushing or jostling during emergencies, while 66.8% expressed unwillingness to assist others. These behaviors underscore the importance of incorporating psychological and behavioral insights into emergency planning. Urban infrastructure designers and policymakers should take these tendencies into account when developing response protocols. Further research is needed to inform targeted interventions that reduce self-preserving behavior and promote collective safety during crises.

**Figure 3:**
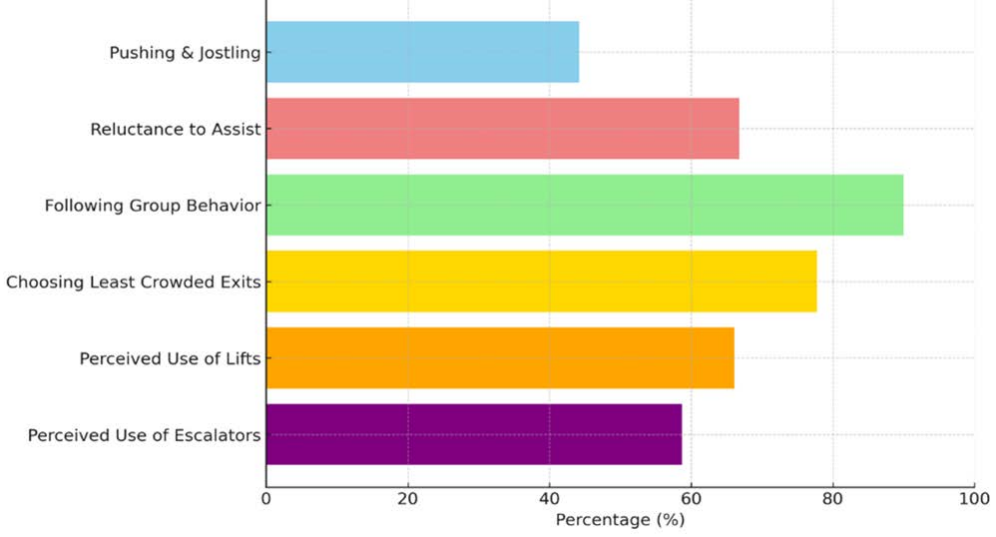
Key behavioral patterns during emergency evacuations

 The empirical findings show that over 90% of participants reported a preference to follow other passengers during emergency evacuations (Figure 3), reflecting a strong reliance on group behavior rather than independent decision-making. Additionally, 77.8% indicated a reluctance to choose the least crowded exit, possibly due to perceived risks or limited visibility. This pattern reflects high symmetry breaking behavior, where individual decisions are shaped by surrounding social cues rather than systematic evaluation. Such behavior may compromise evacuation efficiency by leading to underutilization of available exits.^69^

 The architectural characteristics of the TGB may have significantly shaped participants’ behavioural responses during the simulated evacuation scenario. The bazaar’s narrow corridors, maze-like structure, and limited signage likely increased feelings of spatial constraint and perceptual uncertainty, prompting more reactive and competitive behaviours.^70^ These features can also explain the relatively high incidence of symmetry-breaking strategies, as participants attempted to find less congested pathways in an asymmetric and unfamiliar environment.^71^ In contrast to more open or clearly structured public spaces, the TGB’s complex layout may have reduced opportunities for coordination and cooperation, reinforcing individualized rather than collective responses to perceived danger.

 We also identify those men can exhibit greater instances of “symmetry breaking behavior” than women. This finding aligns with the existing literature which argues that women generally exhibit higher levels of conformity in various behavioral contexts.^72^ Nonetheless, gender demonstrates no correlation with the observed behavior and strategy in this study. Furthermore, individuals with a university education and those over the age of 30 displayed more pronounced symmetry breaking behavior. This suggests that higher educational attainment and age may be associated with a greater likelihood of deviating from uniform behavior patterns during emergency situations.

 The use of escalators and other reliable vertical transport systems can significantly improve evacuation efficiency during emergencies by enabling rapid and orderly movement. However, to maximize their effectiveness and minimize associated risks, urban infrastructure managers and policymakers must ensure that staff are well trained in the operational and safety procedures of these systems. Comprehensive preparedness is essential for the safe deployment of such technologies in critical situations.^73^ As shown in Table 5, a large proportion of participants reported low perceived likelihood of using lifts and escalators during emergencies. Specifically, 66.1% rated the use of lifts as unlikely or very unlikely, and 58.7% expressed similar views about escalators. This perception may stem from concerns over safety, such as the risk of power failure, mechanical malfunction, or overcrowding. The findings indicate a general lack of trust in these systems during critical situations, which should be addressed in future infrastructure planning and public education initiatives.^74^ Moreover, this skepticism might be influenced by past incidents or general safety guidelines that recommend avoiding these systems during emergencies.^28^ These insights highlight a need for further examination into the factors contributing to these perceptions and underscore the importance of improving public education and training about the safe use of vertical transportation systems during emergencies.

**Table 5:** Multi-dimensional framework for emergency evacuation behavior

**Behavior/Observation**	**Percentage**	**Key Insight**	**Implications**
Pushing & jostling behavior	44.2%	Common in high-stress evacuation situations.	Interventions needed to minimize aggressive behavior.
Reluctance to assist others	66.8%	Majority show low inclination to assist.	Policies should encourage cooperative behavior.
Following group behavior	> 90%	Tendency to follow others rather than make independent decisions.	Critical to understand and guide crowd dynamics.
Choosing least crowded exits	77.8%	Preference to avoid crowded exits despite potential risks.	Highlights high symmetry breaking behavior.
Symmetry breaking by gender	Men > Women	Men show greater deviation from uniform behavior.	Requires gender-specific strategies.
Symmetry breaking by education/age	Higher in university-educated and 30 + individuals.	Education and age impact decision-making in emergencies.	Tailored interventions based on demographic insights.
Perceived use of lifts in emergencies	66.1% (low/very low)	Doubts about elevator safety and reliability.	Need for education and trust-building.
Perceived use of escalators	58.7% (low/very low)	Concerns about escalator use during emergencies.	Training and awareness on vertical transportation systems.

 This study highlights several practical recommendations to enhance emergency evacuation management. Training programs should promote cooperative behavior and raise awareness about the risks associated with competitive actions. Urban planners are advised to improve exit visibility and accessibility to reduce symmetry-breaking behavior and prevent crowding at specific points. Regular, high-pressure evacuation drills can further strengthen both individual and collective preparedness. These strategies are especially vital in complex environments like the TGB, where dense and diverse populations require tailored safety solutions.

 To translate these findings into effective action, policymakers and urban planners should develop targeted interventions based on observed behavioral patterns. For instance, placing visual and auditory cues at key locations can reduce symmetry-breaking behavior by directing people toward less congested exits. Additionally, promoting cooperative behavior through routine training drills and public awareness efforts can help minimize competitive actions in crowded settings. When implemented effectively, these strategies can substantially improve both the efficiency and safety of emergency evacuations.

## Study Limitations and Future Research Directions

 While this study provides valuable insights, several limitations should be acknowledged. First, reliance on self-reported data may introduce response biases, as participants may not fully recall or accurately represent their behaviour in hypothetical scenarios. To mitigate this, future studies could incorporate observational methods, video analysis, or wearable biometric devices to obtain more objective behavioural data. Second, the findings are context-specific to the TGB and may not be directly generalizable to other environments. Addressing this limitation would require conducting comparative studies across a variety of architectural and cultural settings to assess the robustness and transferability of the results.

 The study employed a questionnaire-based approach to gather behavioral insights. While this method effectively captures perceptions and intended actions, it may not fully reflect actual behaviors during real emergencies. Individuals often overestimate their willingness to act cooperatively or underreport competitive behaviors due to social desirability bias.^75^ To further enhance the validity of evacuation behavior research, future studies should consider combining self-reported data with objective observational methods. For instance, the use of closed-circuit television (CCTV) footage or overhead video recordings in public spaces can offer direct behavioral evidence to validate or contrast participants’ survey responses. This triangulation approach would reduce reliance on memory-based reporting and allow researchers to identify potential discrepancies between perceived and actual behaviors, providing a more nuanced understanding of decision-making during emergencies.

 The research was conducted in the TGB, a unique high-density market with specific cultural, architectural, and demographic characteristics. While these findings are valuable for traditional marketplaces and similar environments, they may not be directly transferable to other settings, such as airports, train stations, or shopping malls, which have different spatial layouts and emergency response mechanisms. Future research should expand this research to multiple case studies in diverse public spaces would enhance comparability and generalizability.

 The study primarily focused on observable evacuation behaviors and demographic influences. However, it did not explore psychological and emotional factors that might influence decision-making under stress. Future studies should incorporate psychometric assessments, stress-level monitoring, or experimental simulations to assess the impact of panic and cognitive biases on evacuation behaviors.

 This study identified herding tendencies but did not assess the role of authority figures, suggesting future research should examine how leadership and group structures influence evacuation using experiments or agent-based simulations. As a cross-sectional survey, it captured behaviors at a single point in time; longitudinal studies could reveal how experience, training, or prior emergencies shape behavioral adaptation. The findings provide a basis for agent-based modelling of evacuation in traditional marketplaces, enabling realistic simulations that consider spatial constraints, crowd behavior, and strategy variations to improve planning and training in culturally specific contexts.

 Future research should consider conducting similar investigations across diverse cultural and geographical contexts to examine how evacuation behaviours are shaped by local norms, social structures, and spatial layouts. Comparative studies could help determine the extent to which the patterns observed in this study are universal or culturally specific. Moreover, integrating biometric data, such as heart rate variability, galvanic skin response, or eye-tracking, can enrich our understanding of the emotional and cognitive processes underlying evacuation decisions. Such multi-dimensional approaches would offer deeper insights into real-time human behaviour in emergencies and improve the design of culturally responsive evacuation systems.

## Conclusion

 Ensuring safety during emergencies in crowded settings like shopping malls remains a persistent challenge. While prior studies have focused on mathematical modeling and survey analyses in residential and commercial buildings, behavioral responses in real emergencies are still underexplored. This study investigated four key behavioral dimensions including proactive versus reactive strategies, cooperative versus competitive responses, symmetry breaking, and route selection based on responses from 749 participants at the TGB.

 Findings revealed a strong preference for proactive and competitive behaviors, a tendency to follow the crowd, and reluctance to use less visible exits or vertical transport systems. Demographic variables such as age, education, and marital status significantly influenced responses, while gender showed no meaningful correlation. These patterns highlight the importance of designing emergency strategies that account for behavioral tendencies, crowd influence, and demographic differences.

 While self-report bias remains a limitation, the study provides valuable insights to support better emergency preparedness, targeted interventions, and the development of more realistic evacuation models. Future research should incorporate observational and experimental data to improve behavioral accuracy in high-risk scenarios.

 In addition, this study represents one of the first empirical analyses of evacuation behavior within the culturally and architecturally unique context of a traditional marketplace. Unlike previous studies that rely heavily on simulation models or modern built environments, our research provides real-world behavioral data from a historic, crowded, and spatially complex setting. These contributions offer new directions for culturally sensitive emergency planning, particularly in heritage-rich urban areas across the Middle East and beyond.

 In summary, the study emphasizes the importance of integrating behavioral insights into emergency management frameworks. By addressing demographic-specific tendencies and designing environments that facilitate cooperative and efficient evacuations, policymakers can significantly improve safety outcomes in high-density public spaces such as the TGB.

## Competing Interests

 The authors declare no conflicts of interest in relation to this study.

## Ethical Approval

 This study was approved by the Ethics Committee of Tabriz University of Medical Sciences (IR.TBZMED.REC.1396.1145).

## References

[1] Assari A, Mahesh TM, Emtehani MR, Assari E (2011). Comparative sustainability of bazaar in Iranian traditional cities: case studies in Isfahan and Tabriz. Int J Tech Phys Probl Eng.

[2] Ahour I (2011). The qualities of Tabriz historical bazaar in urban planning and the integration of its potentials into megamalls. J Geogr Reg Plann.

[3] Shakib H, Zakersalehi M, Jahangiri V, Zamanian R (2020). Evaluation of Plasco Building fire-induced progressive collapse. Structures.

[4] Fridolf K, Nilsson D, Frantzich H (2013). Fire evacuation in underground transportation systems: a review of accidents and empirical research. Fire Technol.

[5] Shiwakoti N, Tay R, Stasinopoulos P, Woolley PJ. Exploring passengers’ behaviour in an underground train station under emergency condition. In: 38th Australasian Transport Research Forum (ATRF). Melbourne, Australia: Australasian Transport Research Forum; 2016.

[6] Sagun A, Anumba CJ, Bouchlaghem D (2014). Safety issues in building design to cope with extreme events: case study of an evacuation process. J Archit Eng.

[7] Aldahlawi RY, Akbari V, Lawson G (2024). A systematic review of methodologies for human behavior modelling and routing optimization in large-scale evacuation planning. Int J Disaster Risk Reduct.

[8] Liu S, Lo S, Ma J, Wang W (2014). An agent-based microscopic pedestrian flow simulation model for pedestrian traffic problems. IEEE trans Intell Transp Syst.

[9] Choi J, Lee J. Micro-level emergency response: 3d geometric network and an agent-based model. In: Showalter PS, Lu Y, eds. Geospatial Techniques in Urban Hazard and Disaster Analysis. Dordrecht: Springer; 2010. p. 415-29. doi: 10.1007/978-90-481-2238-7_20.

[10] Mirhashemi A, Amirifar S, Tavakoli Kashani A, Zou X (2022). Macro-level literature analysis on pedestrian safety: bibliometric overview, conceptual frames, and trends. Accid Anal Prev.

[11] Haworth B, Usman M, Berseth G, Kapadia M, Faloutsos P (2017). On density-flow relationships during crowd evacuation. Comput Animat Virtual Worlds.

[12] Zhou J, Guo Y, Dong S, Zhang M, Mao T (2019). Simulation of pedestrian evacuation route choice using social force model in large-scale public space: comparison of five evacuation strategies. PLoS One.

[13] Kim S, Park S, Lee JS (2014). Meso-or micro-scale? Environmental factors influencing pedestrian satisfaction. Transp Res D Transp Environ.

[14] Shiwakoti N, Sarvi M, Rose G. Modelling pedestrian behaviour under emergency conditions–state-of-the-art and future directions. In: 31st Australasian Transport Research Forum (ATRF). Gold Coast: ATRF; 2008. p. 457-73.

[15] Macal CM, North MJ. Tutorial on agent-based modeling and simulation. In: Proceedings of the Winter Simulation Conference, 2005. Orlando, FL: IEEE; 2005. p. 14. doi: 10.1109/wsc.2005.1574234.

[16] Varga A. The OMNET + + discrete event simulation system. In: Proceedings of the European Simulation Multiconference (ESM 2001); 2001.

[17] Daamen W, Bovy PH, Hoogendoorn SP. Modelling pedestrians in transfer stations. In: Schreckenberg M, Sharma SD, eds. Pedestrian and Evacuation Dynamics. Duisburg, Germany: Springer; 2002. p. 59-73.

[18] Kobes M, Helsloot I, de Vries B, Post JG (2010). Building safety and human behaviour in fire: a literature review. Fire Saf J.

[19] Kuligowski E (2013). Predicting human behavior during fires. Fire Technol.

[20] Şahin C, Rokne J, Alhajj R (2019). Human behavior modeling for simulating evacuation of buildings during emergencies. Physica A.

[21] Shi L, Xie Q, Cheng X, Chen L, Zhou Y, Zhang R (2009). Developing a database for emergency evacuation model. Build Environ.

[22] Chen J, Yu J, Wen J, Zhang C, Yin Z, Wu J (2019). Pre-evacuation time estimation-based emergency evacuation simulation in urban residential communities. Int J Environ Res Public Health.

[23] Wan X, Li Q, Yuan J, Schonfeld PM (2015). Metro passenger behaviors and their relations to metro incident involvement. Accid Anal Prev.

[24] Shiwakoti N, Tay R, Stasinopoulos P, Woolley PJ (2017). Likely behaviours of passengers under emergency evacuation in train station. Saf Sci.

[25] Mawson AR. Mass Panic and Social Attachment: The Dynamics of Human Behavior. Ashgate Publishing, Ltd; 2012. doi: 10.4324/9781351153201.

[26] Helbing D, Mukerji P (2012). Crowd disasters as systemic failures: analysis of the Love Parade disaster. EPJ Data Sci.

[27] Helbing D, Farkas IJ, Molnar P, Vicsek T. Simulation of pedestrian crowds in normal and evacuation situations. In: Schreckenberg M, Sharma SD, eds. Pedestrian and Evacuation Dynamics. Springer; 2002. p. 21-58.

[28] Ronchi E, Nilsson D (2013). Fire evacuation in high-rise buildings: a review of human behaviour and modelling research. Fire Sci Rev.

[29] Ronchi E, Gwynne S. Computational evacuation modeling in wildfires. In: Encyclopedia of Wildfires and Wildland-Urban Interface (WUI) Fires. Cham: Springer; 2020. p. 115-24.

[30] Averill JD, Mileti DS, Peacock RD, Kuligowski ED, Groner N, Proulx G, et al. Occupant Behavior, Egress, and Emergency Communication. Federal Building and Fire Safety Investigation of the World Trade Center Disaster (NIST NCSTAR 1-7) ***DRAFT for Public Comments***. Gaithersburg, MD: National Construction Safety Team Act Reports (NIST NCSTAR), National Institute of Standards and Technology; 2005. Available from: https://tsapps.nist.gov/publication/get_pdf.cfm?pub_id=909233. Accessed December 20, 2025.

[31] Lyu M, Sun B, Tian X, Wang Y (2023). How does social capital influence shadow evacuation behavior under rainstorm disaster in China. Saf Sci.

[32] Helbing D, Farkas I, Vicsek T (2000). Simulating dynamical features of escape panic. Nature.

[33] Li Q, Zhao M, Zhang Z, Li K, Chen L, Zhang J (2023). Improved social force model considering the influence of COVID-19 pandemic: pedestrian evacuation under regulation. Appl Math Model.

[34] Drury J, Cocking C, Reicher S (2009). The nature of collective resilience: survivor reactions to the 2005 London bombings. Int J Mass Emerg Disasters.

[35] Bode NW, Kemloh Wagoum AU, Codling EA (2014). Human responses to multiple sources of directional information in virtual crowd evacuations. J R Soc Interface.

[36] Sikora W, Malinowski J, Kupczak A. Model of skyscraper evacuation with the use of space symmetry and fluid dynamic approximation. In: Wyrzykowski R, Dongarra J, Karczewski K, Waśniewski J, eds. Parallel Processing and Applied Mathematics. Berlin: Springer; 2012. p. 570-7. doi: 10.1007/978-3-642-31500-8_59.

[37] Tsurushima A. Symmetry breaking in evacuation exit choice: impacts of cognitive bias and physical factor on evacuation decision. In: van den Herik J, Rocha A, Steels L, eds. Agents and Artificial Intelligence. Cham: Springer International Publishing; 2019. p. 293-316. doi: 10.1007/978-3-030-37494-5_15.

[38] Lovreglio R, Fonzone A, dell’Olio L, Borri D, Ibeas A (2014). The role of herding behaviour in exit choice during evacuation. Procedia Soc Behav Sci.

[39] Ji Q, Xin C, Tang SX, Huang JP (2018). Symmetry associated with symmetry break: revisiting ants and humans escaping from multiple-exit rooms. Physica A.

[40] Yu H, Zhou X, Li M, Jiang N, Jia X, Yang L (2024). Experimental study on the movement characteristics of pedestrians in asymmetric merging structures. J Build Eng.

[41] Lin J, Zhu R, Li N, Becerik-Gerber B (2020). Do people follow the crowd in building emergency evacuation? A cross-cultural immersive virtual reality-based study. Adv Eng Inform.

[42] Moussaïd M, Helbing D, Theraulaz G (2011). How simple rules determine pedestrian behavior and crowd disasters. Proc Natl Acad Sci U S A.

[43] Parisi DR, Dorso CO (2005). Microscopic dynamics of pedestrian evacuation. Physica A.

[44] Li R, Wang X, Lovreglio R, Ding H, Wang Q, Chen J (2025). Influence of subsequent path and pressure on pedestrian route choice in emergency evacuations. Physica A.

[45] Kinateder M, Warren WH (2021). Exit choice during evacuation is influenced by both the size and proportion of the egressing crowd. Physica A.

[46] Cuesta A, Abreu O, Alvear D. Future challenges in evacuation modelling. In: Cuesta A, Abreu O, Alvear D, eds. Evacuation Modeling Trends. Cham: Springer International Publishing; 2016. p. 103-29. doi: 10.1007/978-3-319-20708-7_5.

[47] Bakhshian E, Martinez-Pastor B (2023). Evaluating human behaviour during a disaster evacuation process: a literature review. J Traffic Transp Eng (Engl Ed).

[48] Wang Y, Kyriakidis M, Dang VN (2021). Incorporating human factors in emergency evacuation – an overview of behavioral factors and models. Int J Disaster Risk Reduct.

[49] Russo F, Rindone C (2024). Methods for risk reduction: training and exercises to pursue the planned evacuation. Sustainability.

[50] Marsousi N, Khani MB (2011). The study of economic function of Tabriz bazaar and its surrounding areas. Hum Geogr Res.

[51] Franke TM, Ho T, Christie CA (2011). The chi-square test: often used and more often misinterpreted. Am J Eval.

[52] Andridge RR, Little RJ (2010). A review of hot deck imputation for survey non-response. Int Stat Rev.

[53] Ekenga CC, Ziyu L (2019). Gender and public health emergency preparedness among United States adults. J Community Health.

[54] Mortazavi S, Poelker KE. Women in Iran. In: Brown CM, Gielen UP, Gibbons JL, Kuriansky J, eds. Women’s Evolving Lives: Global and Psychosocial Perspectives. Cham: Springer International Publishing; 2017. p. 73-90. doi: 10.1007/978-3-319-58008-1_5.

[55] Eagly AH, Wood W (1999). The origins of sex differences in human behavior: evolved dispositions versus social roles. Am Psychol.

[56] Fothergill A (1996). Gender, risk, and disaster. Int J Mass Emerg Disasters.

[57] Akbarzadeh O, Golzad H, Moshashaei P (2025). Health promotion model insights on determinants of personal protective equipment use in occupational settings. Iran J Health Educ Health Promot.

[58] Fridolf K, Nilsson D, Frantzich H (2013). Fire evacuation in underground transportation systems: a review of accidents and empirical research. Fire Technol.

[59] Albis KA, Radhwi MN, Gawad AF (2015). Fire dynamics simulation and evacuation for a large shopping center (mall): part I, fire simulation scenarios. Am J Energy Eng.

[60] Ahn C, Kim J, Lee S (2016). An analysis of evacuation under fire situation in complex shopping center using evacuation simulation modeling. Procedia Soc Behav Sci.

[61] Mohd Ibrahim A, Venkat I, De Wilde P, Mohd Romlay MR, Bahamid A (2022). The role of crowd behavior and cooperation strategies during evacuation. Simulation.

[62] Cheng Y, Zheng X (2018). Can cooperative behaviors promote evacuation efficiency?. Physica A.

[63] Brown R (2000). Social identity theory: past achievements, current problems and future challenges. Eur J Soc Psychol.

[64] Tyler TR, Blader SL (2001). Identity and cooperative behavior in groups. Group Process Intergroup Relat.

[65] Gillies RM (2000). The maintenance of cooperative and helping behaviours in cooperative groups. Br J Educ Psychol.

[66] Nettle D, Colléony A, Cockerill M (2011). Variation in cooperative behaviour within a single city. PLoS One.

[67] Westermann O, Ashby J, Pretty J (2005). Gender and social capital: the importance of gender differences for the maturity and effectiveness of natural resource management groups. World Dev.

[68] Kis EE, Deaconu LT, Roman E, Ştefănescu L, Meltzer M, Pop C (2013). Assessment of population awareness and preparedness level regarding the environmental emergency situations. Adv Environ Sci.

[69] Zhu KJ, Shi Q (2016). Experimental study on choice behavior of pedestrians during building evacuation. Procedia Eng.

[70] Pourjafar MR, Pourjafar A (2016). Iranian Islamic urban design features of Tabriz; case study: great bazaar of the city. Cult Islam Archit Urban.

[71] Montello DR, Davis RC, Johnson M, Chrastil ER (2023). The symmetry and asymmetry of pedestrian route choice. J Environ Psychol.

[72] Eagly AH, Chrvala C (1986). Sex differences in conformity: status and gender role interpretations. Psychol Women Q.

[73] Bukowski RW (2012). Addressing the needs of people using elevators for emergency evacuation. Fire Technol.

[74] Lee HY, Lee SH, Hong WH (2014). A study on the escalator evacuation model using the buildingEXODUS. Journal of the Architectural Institute of Korea Planning & Design.

[75] Cox TH, Lobel SA, McLeod PL (1991). Effects of ethnic group cultural differences on cooperative and competitive behavior on a group task. Acad Manage J.

